# miR-506 Regulates Epithelial Mesenchymal Transition in Breast Cancer Cell Lines

**DOI:** 10.1371/journal.pone.0064273

**Published:** 2013-05-22

**Authors:** Himanshu Arora, Rehana Qureshi, Woong-Yang Park

**Affiliations:** 1 Department of Biomedical Sciences, Seoul National University College of Medicine, Seoul, Korea; 2 Department of Molecular Cell Biology, Sungkyunkwan University School of Medicine, Seoul, Korea; 3 Samsung Genome Institute, Samsung Medical Center, Seoul, Korea; Wayne State University School of Medicine, United States of America

## Abstract

Epithelial-mesenchymal transition (EMT) is an important parameter related to breast cancer survival. Among several microRNAs predicted to target EMT-related genes, miR-506 is a novel miRNA found to be significantly related to breast cancer patient survival in a meta-analysis. miR-506 suppressed the expression of mesenchymal genes such as Vimentin, Snai2, and CD151 in MDA-MB-231 human breast cancer cell line. Moreover, NF**-**κB bound to the upstream promoter region of miR-506 to suppress transcription. Overexpression of miR-506 inhibited TGFβ-induced EMT and suppressed adhesion, invasion, and migration of MDA-MB-231 cells. From these results, we concluded that miR-506 plays a key role in the process of EMT through posttranslational control of EMT-related genes.

## Introduction

Epithelial to mesenchymal transition (EMT) is a process that enables cancer cells to lose their cell-cell and cell-matrix contacts, gain migratory properties, and become motile mesenchymal cells [1]. Transcriptional reprogramming allows epithelial tumor cells to lose cell polarity and cell-junction proteins while acquiring the signal transduction activities associated with mesenchymal cells that facilitate migration and survival in an anchorage-independent environment [2]. The gain of mesenchymal cell markers such as Vimentin (VIM), Snail homolog 2 (SNAI2), and fibronectin (FN) has been observed in tumor progression [3], [4]. In addition, cellular changes resulting in a more mesenchymal-like state are associated with poor prognosis.

Mesenchymal-like tumor cells gain migratory capacity through abnormal survival signals via receptors such as fibroblast growth factor receptor (FGFR), hepatocyte growth factor receptor (MET), transforming growth factor beta receptors (TGFβRs), insulin like growth factor 1 receptor (IGF1R), platelet derived growth factor receptor (PDGFR), and regulatory kinases such as phosphoinositide-3-kinase (PI3K), v-akt murine thymoma viral oncogene (AKT), and mechanistic target of rapamycin (mTOR) [5]–[10].

Nuclear factor of kappa light polypeptide gene enhancer in B-cells (NF**-**κB) is upregulated in human breast tumor cell lines, carcinogen transformed mammary epithelial cells, the majority of primary human and rodent breast tumor tissue samples [11]. It has been reported to be a central mediator of EMT in a mouse model of breast cancer progression [12], [13]. More recently, NF**-**κB was shown to act upstream of SNAI2 expression during EMT of MCF10A human mammary epithelial cells overexpressing a constitutively active IGF1R [14]. SNAI2 can also repress endogenous E-cadherin (CDH1) gene expression. In breast cancer cell lines, SNAI2 levels were shown to correlate with loss of E-cadherin transport [15].

Proteins of the tetraspanin family form complexes with integrins and function in cell-cell adhesion in a cadherin-independent manner. CD151 is the first of 33 tetraspanin family members associated with promotion of metastasis [16]. In cancer cells, CD151 regulates adhesion-dependent signaling and post-adhesion events, including cell migration [17], [18]. CD151 also plays a role in the induction of EMT and the overall survival of patients with cancer [16], [19]. TGFβ has been postulated to be a pro-oncogenic factor acting late in tumor progression. In transformed cells, TGFβ enhances crucial metastatic processes, including the ability to degrade the extracellular matrix, cell invasiveness, and epithelial-mesenchymal transition [20]. NF**-**κB and CD151 induction are required for the TGFβ-mediated response [20], [21].

Here, we used enrichment analysis to identify miR-506 as being a miRNA that targets the 3′ untranslated regions (UTRs) of EMT-related genes such as SNAI2, VIM, and CD151. Importantly, we found an NF**-**κB binding motif upstream of the promoter region of miR-506. We therefore hypothesized that NF**-**κB binds upstream of the promoter region of miR-506, which further targets the 3′UTR of CD151 and other EMT markers such as VIM and SNAI2 to regulate EMT.

## Results

### Selection of miRNAs Regulating EMT and Survival of Patients with Breast Cancer

**Figure 1:**
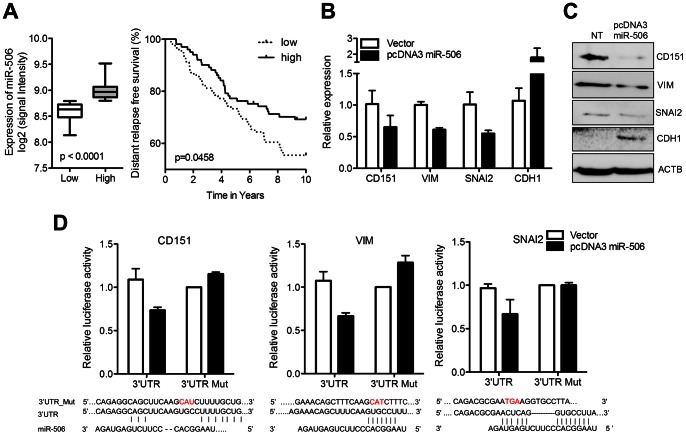
Selection of miR-506 as a candidate EMT-regulating miRNA and suppression of EMT-related genes by miR-506. (A) The clinical significance of miR-506 in patients with breast cancer. Tissue samples from patients with breast cancer were analyzed GEO dataset, and patients were divided into two sets based on miRNA expression (greater or less than the median, p<0.05). P values were computed by log rank and Wilcoxon tests of significance. (B) Expression of CD151, VIM, SNAI2, and CDH1 in the presence of overexpression of miR-506 was determined by qRT-PCR and (C) western blotting. (D) miR-506 targets the 3′UTRs of CD151, VIM, and SNAI2. The 3′ UTRs of CD151, VIM, and SNAI2 were cloned into psiCHECK2 vector. Mutations in the miR-506 target sites in these UTRs were generated. MDA-MB-231 cells were cotransfected with UTR (normal and mutant)-bearing psiCHECK2 vector and pcDNA3-cloned miR-506 for 48 hrs. Luciferase assays were performed. Renilla expression was normalized to the luciferase gene on the psiCHECK2 vector. All experiments were performed in triplicate and repeated at least twice.

We previously investigated the function of miRNAs in tumor initiation, progression, and metastasis [22], [23]. In this study, we aimed to investigate the role of miRNAs in EMT. We therefore considered genes related to the regulation of EMT from the Gene Ontology database (GO:0001837) and other independent studies. miRNAs predicted to target these genes were selected using TargetScan. Interestingly, we found that miR-506 was predicted to target 16 EMT-related genes (Table S2). We performed a meta-analysis of a publically available human breast cancer miRNA expression database (GSE22216) [24] and investigated the effects of miR-506 on distant-relapse-free survival (DRFS) in breast cancer. The patient samples were divided into high and low miR-506 based on median expression levels (P<0.05, n = 206). miR-506 showed a significant association with DRFS (p = 0.0458) in more than 98% of patients (Fig. 1A), while miR-124, which targets the same seed sequences, showed no significant impact on DRFS in the same patients (data not shown), thereby leading us to further investigate miR-506.

### Regulation of EMT-related Genes by miR-506

miR-506 is predicted to target the 3′UTRs of CD151, VIM, and SNAI2. We overexpressed precursor miR-506 in MDA-MB-231 human breast cancer cell lines to monitor the expression of these target genes and CDH1 (an epithelial marker). The expression levels of CD151, VIM, and SNAI2 were suppressed by miR-506, while CDH1 was upregulated at mRNA (Fig. 1B) and protein level (Fig. 1C). In addition, we also observed the effects of miR-506 on EMT-related genes in another breast cancer cell line, MDA-MB-468 by real-time reverse transcription polymerase chain reaction (RT-PCR) (Fig. S1). We cloned miR-506-binding sequences in the 3′UTR of CD151, VIM, and SNAI2 for luciferase assay. Overexpression of miR-506 resulted in a significant decrease in luciferase activity with the wildtype 3′UTR of CD151, VIM, and SNAI2, but not with mutant 3′UTR sequences (Fig. 1D). Overexpression of miR-506 also induced morphological changes in MDA-MB-231 cells from long and elongated mesenchymal-like cell to round and circular epithelial-like cell (Fig. S2).

### NF-κB-mediated Regulation of miR-506

**Figure 2:**
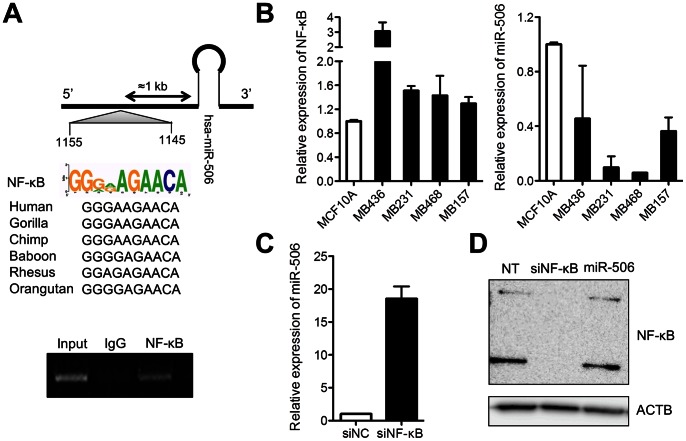
Binding of NF-κB to upstream sequences of miR-506 to suppress transcription. (A) Chromatin immunoprecipitation showing the interaction between NF-κB and miR-506. (B) miR-506 and NF-κB correlation in different breast cancer cell lines. (C) Expression of miR-506 in MDA-MB-231 cells transfected with siNF-κB. (D) NF-κB expression in MDA-MB-231 cells transfected with miR-506.

Promoter sequence analysis revealed a putative NF**-**κB binding site at −1013 bp from precursor miR-506. NF**-**κB is a well-known regulator of EMT for the positive correlation with several EMT markers in patients with breast cancer (Fig. S3). We confirmed binding of NF**-**κB upstream of miR-506 by chromatin immunoprecipitation (ChIP) assay (Fig. 2A). We also investigated the correlation between NF-κB mRNA expression and miR-506 in MDA-MB-436, MDA-MB-231, MDA-MB-468, and MDA-MB-157 cells. In comparison to MCF10A, normal breast epithelial cells, the expression of NF**-**κB was consistently high and miR-506 level was low in the four cancer cell lines. On the other hand, the expression of NF**-**κB mRNA was low and the miR-506 level was high in MCF10A cells (Fig. 2B). To test whether NF**-**κB suppressed miR-506, we knocked down NF**-**κB in MDA-MB-231 cells using siNF**-**κB. As shown in Fig. 2C, miR-506 expression was induced by the suppression of NF**-**κB. When we overexpressed miR-506 in MDA-MB-231 cells, the expression of NF**-**κB was not changed (Fig. 2D).

### Regulation of Epithelial to Mesenchymal Transition by miR-506

**Figure 3:**
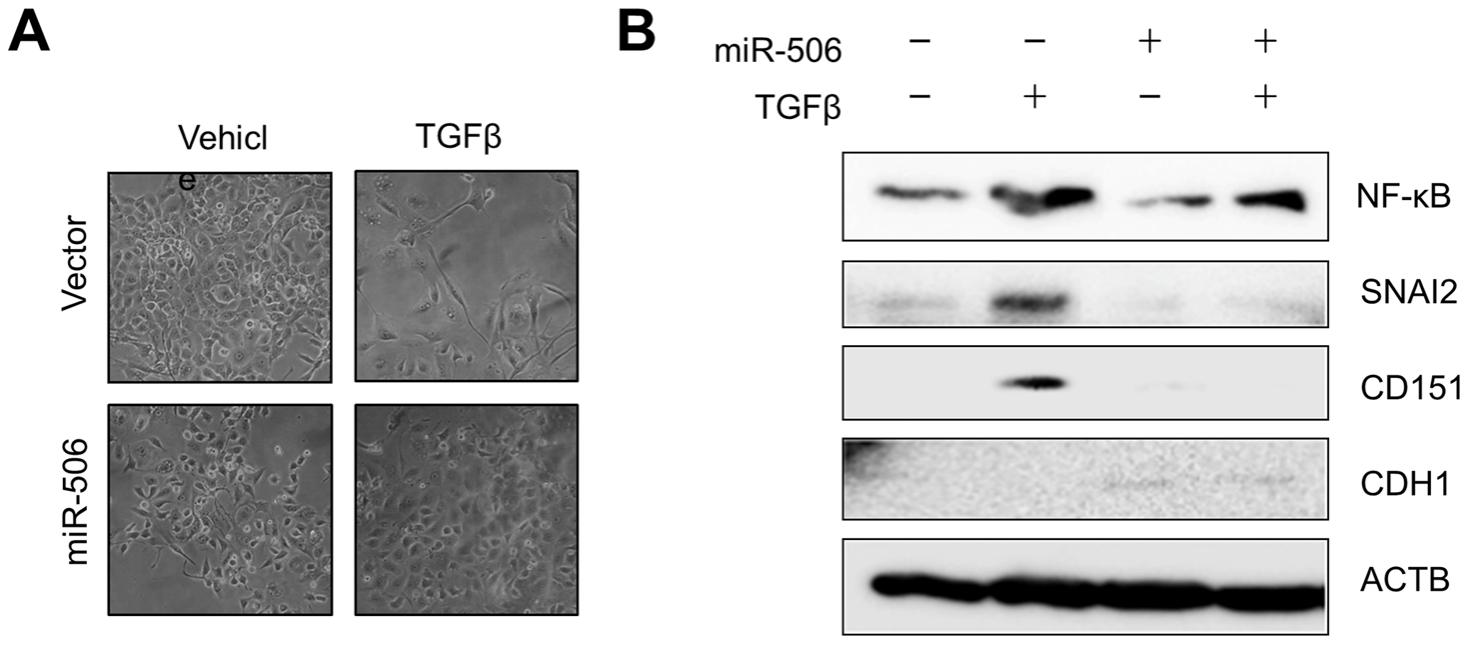
Regulation of epithelial to mesenchymal transition by miR-506. (A) miR-506 could induce TGFβ-induced morphological changes in MCF10A cells. (B) Expression of various EMT markers such as SNAI2, CD151, CDH1, and NF**-**κB were checked in miR-506 in TGFβ-treated MCF10A cells.

Our results indicated that NF-κB bound to the promoter region of miR-506, which itself targets several EMT markers in an inverse manner. We wanted to know if miR-506 suppression was necessary for NF-κB to regulate EMT. In order to understand this, we used TGFβ as an inducer of EMT [25], [26]. We investigated the effect of miR-506 overexpression on TGFβ-induced EMT in MCF10A cells. We found that overexpression of miR-506 inhibited morphological changes in TGFβ-treated MCF10A cells (Fig. 3A), whereas in the absence of TGFβ treatment, overexpression of miR-506 did not affect epithelial characteristics. The suppressive function of miR-506 in TGFβ-induced EMT was validated by the suppression of expression of EMT-related genes such as SNAI2, CD151, and CDH1 (Fig. 3B). Interestingly, the expression of NF-κB remained unaffected in miR-506-transfected cells induced by TGFβ [27], and remained induced in TGFβ and miR-506 transfected cells as compared to control conditions. These results strongly suggested that miR-506 acted downstream of NF-κB and that NF-κB could not induce EMT in the presence of miR-506 (Fig. 3B).

### Effects of miR-506 in Cell Adhesion, Migration, and Invasion

**Figure 4:**
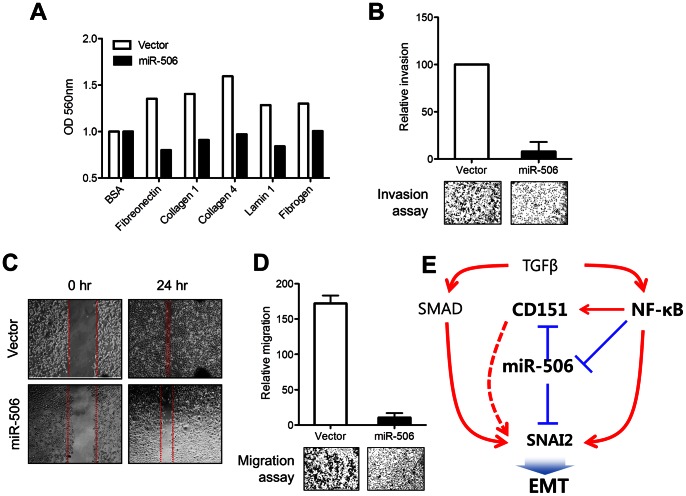
Effects of miR-506 in cell adhesion, migration, and invasion. (A) Overexpression of miR-506 in MDA-MB-231 cells may alter the binding to the various matrix components responsible for cell adhesion (extracellular matrix array) such as fibronectin, collagen 1, collagen 4, lamin 1, fibrogen (bovine serum albumin was used as a negative control). (B) Invasive capacity of MDA-MB-231 cells was analyzed by Matrigen Invasion Assay. Overexpression of miR-506 on the regulation of overall migratory properties were observed by (C) wound healing assay or (D) Boyden Chamber Assay (3D migration assay). (E) Summary of miR-506-mediated control of EMT.

We evaluated the effects of miR-506 on cell adhesion by *in vitro* adhesion assay. The overexpression of miR-506 decreased the adhesion of MDA-MB-231 cells to a range of extracellular matrix components such as fibronectin, collagen 1, collagen 4, laminin1, and fibrogen (Fig. 4A). We also investigated the effects of miR-506 on the invasive and migratory activity of MDA-MB-231 cells in *in vitro* assays. The overexpression of miR-506 in MDA-MB-231 cells was found to suppress invasive potential through Matrigel (Fig. 4B). The migration of MDA-MB-231 cells was also suppressed by the overexpression of miR-506 (Fig. 4C and 4D).

## Discussion

miRNAs are important regulators of gene expression and affect fundamental cellular processes such as differentiation, proliferation, the cell cycle, and apoptosis [28]–[30]. In addition, miRNAs can play a role in cancer processes, including tumorigenesis, invasion, and recurrence [22], [28]–[31]. Recent miRNA research has focused on various high-throughput biochemical screens that measure the expression of over 1,000 human miRNAs in human cancer. Several hundreds of miRNAs were mapped to altered regions in the cancer genome. Either overexpression or deletion of miRNAs can drive the initiation and progression of human cancer. miRNAs also play critical roles in normal stem cell function during development and have emerged as important regulators of cancer stem cells [32]. The modulation of specific miRNA alterations in cancer cells using miRNA replacement or anti-miRNA technologies was shown to restore miRNA activity and repair gene regulatory networks affecting apoptotic signaling pathways or drug sensitivity and to improve treatment outcomes [33]. In this study, we observed that miR-506 played a role as a master suppressor of EMT in breast cancer through the direct targeting CD151, VIM and SNAI2.

The role of NF**-**κB in normal mammary development [34], [35] and its constitutive activation in breast cancer has been well established [34], [36]. NF**-**κB is thought to function in the initiation and growth of cancer cells at sites of metastasis. There is growing evidence that NF**-**κB plays a central role in EMT [37], [38]. There is an NF**-**κB binding site in the promoter region of miR-506, and we confirmed the suppressive function of NF**-**κB on miR-506 expression. In this sense, the suppression of miR-506 by NF**-**κB might be required for the progression of EMT.

Cytokines of the TGFβ superfamily have been implicated in a variety of normal and pathologic phenomena. It is well known that TGFβ induces EMT [25], [26]. In addition, the depletion of CD151 attenuates pulmonary metastasis of breast cancer cells by regulating TGFβ signaling [21]. Since CD151 is positively correlated with TGFβ, we treated MCF10A normal breast cancer cells with TGFβ to investigate the involvement of miR-506 in the regulation of EMT. We found that overexpression of miR-506 suppressed TGFβ-mediated induction of EMT marker. These experiments suggested that miR-506 inhibited TGFβ-induced EMT signaling. We studied the functional relevance of miR-506 with respect to invasion, migration, and adhesion, and showed that overexpressed miR-506 acted as a tumor suppressor miRNA in breast cancer cells. Further studies will be needed to validate the clinical utility of miR-506 as a measure of breast cancer treatment.

## Materials and Methods

### Cell Culture and Cloning

MDA-MB-231, MDA-MB-157, and MDA-MB-468 human breast cancer cell lines were purchased from ATCC and maintained in Dulbecco's modified Eagle's medium (DMEM, Sigma Aldrich, St Louis, MO) supplemented with 10% fetal bovine serum (FBS), 100 U/ml penicillin, and 100 µg/ml streptomycin. MDA-MB-436 cells were maintained in Leibovitz’s L-15 medium supplemented with 10% FBS and 10 µg/ml insulin. MCF10A normal mammary epithelial cells were grown in base medium (MEBM) supplemented with MEGM additives (Lonza Clonetics, Walkersville, MD). MCF10A cells were treated with 20 ng/mlTGF–β1 (R&D System Inc., Minneapolis, MN) for the indicated times to induce EMT. Human pre-miR-506 was amplified and cloned into pcDNA3 (Invitrogen, Carlsbad, CA) using gene-specific primers (Table S1) for human genomic DNA.

### Quantitative Reverse Transcriptase-polymerase Chain Reaction (RT-PCR)

Total RNA was extracted using TRIzol and then reverse transcribed into complementary DNA using Superscript II reverse transcriptase (Invitrogen, Carlsbad, CA) and oligo-(dT) 12–18-mer primers according to the manufacturer’s protocol. Quantitative RT-PCR was performed in a reaction mixture containing SYBR Premix Ex Taq (Takara Bio Inc., Shiga, Japan). Quantitation of microRNAs was performed using TaqMan miRNA assays (Applied Biosystems, Foster City, CA) according to the manufacturer’s protocol. Samples were analyzed using an ABI PRISM 7000 (Applied BioSystems). All PCR reactions were performed in triplicate and the relative quantitative method was applied by using the averaged ΔCt from the untreated cells. The endogenous control was glyceraldehyde 3-phosphate dehydrogenase (GAPDH) or U6B.

### Luciferase Assay

The 3′ UTRs of CD151, VIM, and SNAI2 were fused to the Renilla luciferase gene using the XhoI/NotI restriction sites in the psiCHECK2vector (Promega, Fitchburg, WI). Mutations in the miR-506 target site in these UTRs were generated using the QuikChange Multi Site-directed Mutagenesis kit (Stratagene, La Jolla, CA). Primers used to amplify wild type (WT) and mutant (MUT) 3′UTRs are listed in Table S1. Luciferase assays were performed using the Dual-Luciferase assay (Promega). Renilla expression was normalized to the luciferase gene on the psiCHECK2 vector.

### Western Blotting

Cells were harvested and lysed in RIPA buffer and Protease Inhibitor Cocktail (Sigma). Protein extracts were separated by sodium dodecyl sulfate-polyacrylamide gel electrophoresis (SDS-PAGE), followed by transfer to polyvinylidene fluoride membranes (Bio-Rad, Hercules, CA). Membranes were incubated with CD151 (Santa Cruz Biotechnology Inc., Santa Cruz, CA), CDH1 (Abcam, Cambridge, UK), SNAI2 (Cell Signaling Technology, Danvers, MA), or NF-κB (Santa Cruz Biotechnology Inc.) antibodies in Tris-buffered saline Tween 20 buffer with non-fat dry milk, followed by incubation with horseradish-peroxidase-conjugated secondary antibody (Bio-Rad). Immunoreactive bands were visualized using the Chemiluminescent Substrate Kit (Thermo Scientific, Rockford, IL).

### Adhesion, Invasion and Migration Analysis

Adhesion assays were performed 48 hours after transfection with miR-506 using a Cytoselect 48-well cell adhesion assay (Cells Biolabs, San Diego, CA) according to the manufacturer’s protocol. For invasion assays, cells were seeded in a Matrigel-coated chamber (BD, Franklin, NJ), and viable cells were quantitated after 24, 48, 72, and 96 hours using 3-(4,5 dimethylthiazole-2,5dipheniltetrazolium bromide) (MTT; Sigma). For migration assays, cells were seeded into the upper chamber of a Transwell insert (Corning Life Sciences, Lowell, MA) in serum-free medium 48 hours after transfection. The lower chamber was filled with medium containing 10% FBS. The cells that migrated onto the lower surface of the insert were fixed with 4% formaldehyde and stained with crystal violet (3D migration assay). For the wound healing migration assay (2D migration assay), a wound was made in a cell monolayer and images were captured initially and at regular intervals during cell migration to close the wound. These images were compared to quantify the migration rate of the cells.

### Chip Assay

Chromatin immunoprecipitation was modified from the EZ-CHIP (Upstate, Tenecula, CA) protocol using anti-NF-kB antibody (Santa Cruz Biotechnology Inc.). Input genomic DNA served as a control.

### Bioinformatic and Statistical Analyses

Quantitative results were expressed as mean ± standard error of the mean (s.e.m.). Independent Student's t-tests or Wilcoxon signed-rank tests were performed to analyze gene and miRNA expression levels. The log-rank test was used to test for differences in survival in univariate analysis.

Normalized mRNA and miRNA expression data were obtained from the Gene Expression Omnibus (GEO, http://www.ncbi.nlm.nih.gov/geo/) using series matrix files of dataset GSE2990. Correlation was assessed by Pearson correlation [r], with p<0.05 considered statistically significant. Kaplan-Meier plots were used to estimate distant metastasis-free survival in the GEO dataset GSE22216 [24]. Samples were stratified into high and low miRNA expression groups based on the median expression level. The significance of survival differences between groups was assessed by the log-rank test, with p<0.05 considered statistically significant.

## Supporting Information

Figure S1 **Expression of CD151, VIM, and SNAI2 in miR-506-overexpressed MDA-MB-468 human breast cancer cell lines.**
(DOCX)

Figure S2 **Morphological changes in miR-506-overexpressed MDA-MB-231 human breast cancer cell lines.**
(DOCX)

Figure S3 **Correlation between NF-κB and other epithelial to mesenchymal transition marker genes in breast cancer patients.**
(DOCX)

Table S1 **Primer sequences used for 3′-UTR cloning, RT-PCR, ChIP and expression vector cloning.**
(PDF)

Table S2 **List of epithelial to mesenchymal transition-related target genes for miR-506.**
(PDF)
