# Supplementary figures and images for: miR-506 Regulates Epithelial Mesenchymal Transition in Breast Cancer Cell Lines
Source: PLoS One. 2013 May 22;8(5):e64273. doi: 10.1371/journal.pone.0064273 (PMC3661463; doi:10.1371/journal.pone.0064273)

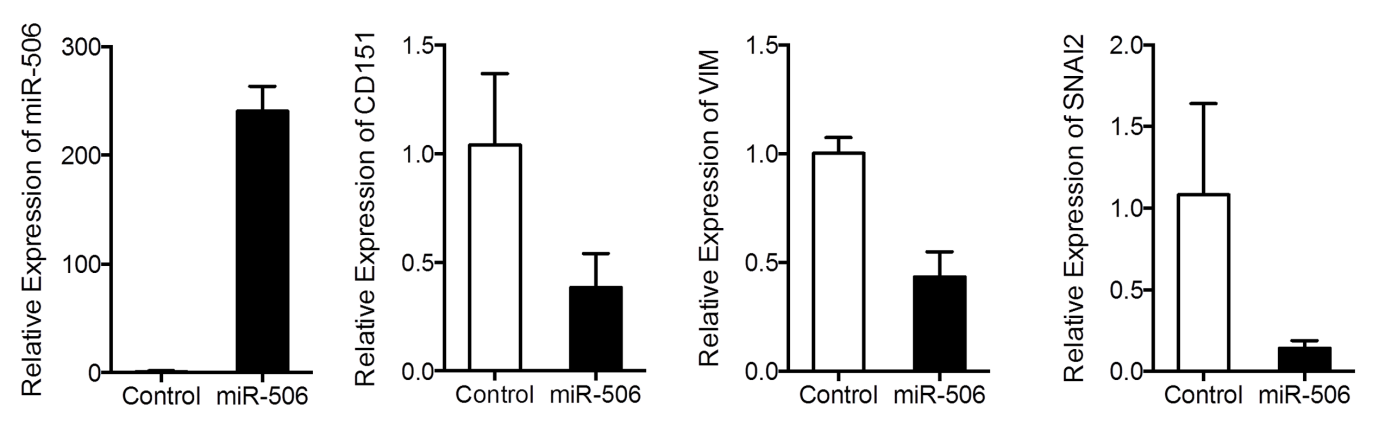


**Fig. S1.** Expression of CD151, VIM, and SNAI2 in miR-506-overexpressed MDA-MB-468 human breast cancer cell lines

Supplement: Figure S1 — Expression of CD151, VIM, and SNAI2 in miR-506-overexpressed MDA-MB-468 human breast cancer cell lines. (DOCX) [file pone.0064273.s001.docx]

**
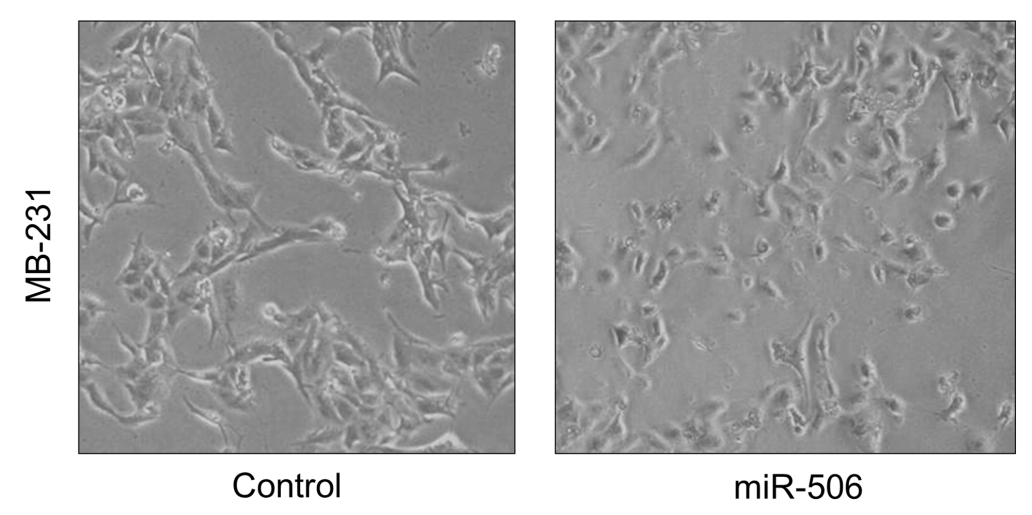
**

**Fig. S2.** Morphological changes in miR-506-overexpressed MDA-MB-231 human breast cancer cell lines.

Supplement: Figure S2 — Morphological changes in miR-506-overexpressed MDA-MB-231 human breast cancer cell lines. (DOCX) [file pone.0064273.s002.docx]
