# Supplementary material for: miR-506 Regulates Epithelial Mesenchymal Transition in Breast Cancer Cell Lines
Source: PLoS One. 2013 May 22;8(5):e64273. doi: 10.1371/journal.pone.0064273 (PMC3661463; doi:10.1371/journal.pone.0064273)
Supplement: Figure S3 — Correlation between NF-κB and other epithelial to mesenchymal transition marker genes in breast cancer patients. (DOCX) [file pone.0064273.s003.docx]

**
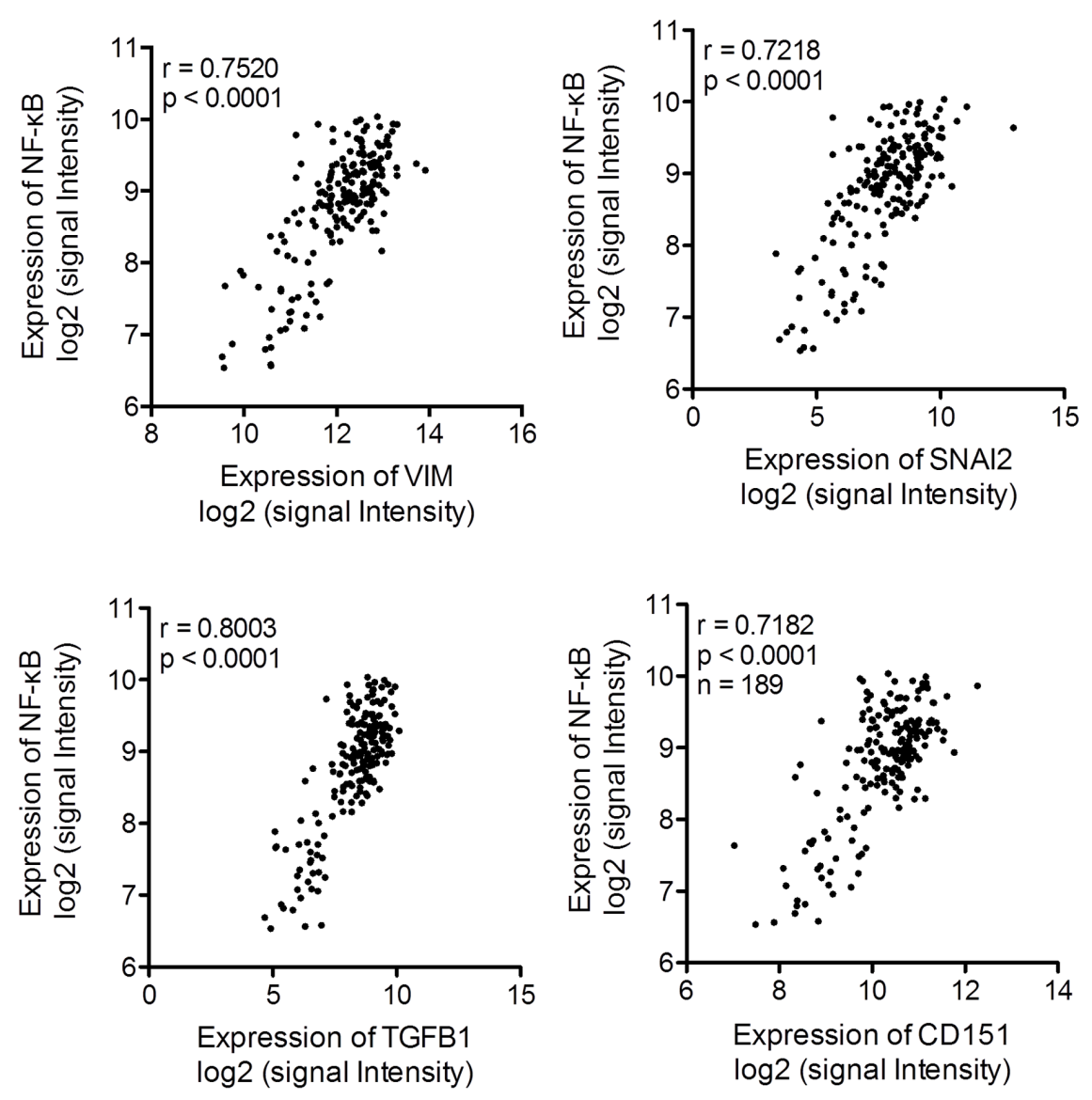
**

**Fig. S3.** Correlation between NF**-**κB and other epithelial to mesenchymal transition marker genes in breast cancer patients.
