# Supplementary material for: miR-506 Regulates Epithelial Mesenchymal Transition in Breast Cancer Cell Lines
Source: PLoS One. 2013 May 22;8(5):e64273. doi: 10.1371/journal.pone.0064273 (PMC3661463; doi:10.1371/journal.pone.0064273)
Supplement: Table S1 — Primer sequences used for 3′-UTR cloning, RT-PCR, ChIP and expression vector cloning. (PDF) [file pone.0064273.s004.pdf]

**Supplementary Table S1.** Primer sequences used for 3’-UTR cloning, RT-PCR, ChIP and expression vector cloning.

| Gene Name | Forward | Reverse |
|---|---|---|
| 3' UTR cloning | | |
| CD151, wild type | AATTCTCGAGCACTACTGACCCTGCCTTGG | AATTGCGGCCGCCAGGCAGAGAGTGTGTCTGG |
| SNAI2, wild type | AATTCTCGAGTTTGCTGATGGCTAGATTGAGA | AATTGCGGCCGCTTCAACAATGGCAACCAGAC |
| VIM, wild type | AATTCTCGAGTCCAAACTTTTCCTCCCTGA | AATTGCGGCCGCTCTTGCGCTCCTGAAAAACT |
| CD151, mutant | GAGGCAGCTTCAAGCATCTTTTGCTGCGCAC | GTGCGCAGCAAAAGATGCTTGAAGCTGCCTC |
| SNAI2, mutant | GAGATCTGCCAGACGCGAATGAAGGTGCCTTAAAAAGTATTC | GAATACTTTTTAAGGCACCTTCATTCGCGTCTGGCAGATCTC |
| VIM, mutant | GAAACAGCTTTCAAGCATCTTTCTGCAGTTTTTCAGG | CCTGAAAAACTGCAGAAAGATGCTTGAAAGCTGTTTC |
| RT-PCR | | |
| CD151 | AACCTCAGAGGCAGCTTCAA | GACCACCAGGCAGAGAGTGT |
| SNAI2 | CAAGAACAAAACACAGGAGAATG | GCCAGGAATGTTCAAAGCTAA |
| VIM | TTCTCAGCATCACGATGACC | TCTTGCGCTCCTGAAAAACT |
| ChIP | | |
| miR-506 | TCAAATCAGGGATTTAGTATTTTCA | TTTATGGGTACAGAGTCACAGTTAGG |
| Expression Vectors | | |
| miR-506 | CCGGAATTCTTGCACCTTTTGGAGTGAAA | ATTGCGGCCGCACCTGGAAATGGCTCATCAC |
