# Supplementary material for: miR-506 Regulates Epithelial Mesenchymal Transition in Breast Cancer Cell Lines
Source: PLoS One. 2013 May 22;8(5):e64273. doi: 10.1371/journal.pone.0064273 (PMC3661463; doi:10.1371/journal.pone.0064273)
Supplement: Table S2 — List of epithelial to mesenchymal transition-related target genes for miR-506. (PDF) [file pone.0064273.s005.pdf]

Supplementary Table S2. List of epithelial to mesenchymal transition-related target genes for miR-506.

Gene	conserved sites				Total Context Score	Aggregate PCT
	Total	8mer	7mer-m8	7mer-1A		
SNAI2	3	1	2	0	-0.71	0.83
FOXQ1	1	1	0	0	-0.4	0.87
VIM	1	0	1	0	-0.26	0.85
CDH2	1	0	1	0	-0.28	0.82
ITGA7	1	1	0	0	-0.32	0.92
ITGB1	2	0	2	0	-0.56	0.97
ROCK1	1	1	0	0	-0.32	0.84
CD151	1	0	1	0	-0.21	0.89
TCF3	1	0	0	1	-0.14	0.89
CAV1	1	0	1	0	-0.22	0.87
AKT2	1	1	0	0	-0.09	0.96
SMAD4	3	0	2	1	-0.06	> 0.99
WIPF1	1	1	0	0	-0.32	0.94
ELK3	1	0	1	0	-0.37	0.89
ETS1	1	1	0	0	-0.08	0.81
COL41	1	1	0	0	-0.5	0.93
